# Analysis of the Effect of Nine Consecutive Years' Intensive Management and Number of Times Achieving the Target Control on Endpoint Events in T2DM Patients in Sanlitun Community Health Service Center in Beijing

**DOI:** 10.1155/2020/3646342

**Published:** 2020-02-20

**Authors:** Chen-Mei Zhao, Xue-Li Cui, Gang Wan, Yu-Zhe Lu, Yu-Qin Niu, Cheng-Yu Su, Shuo Cao, Guan-Xiu Liang, Hong-Wei Chen, Jing Li, Xia Lu, Zhi-Yun Deng, Xue-Hui Yu, Wen-Xia Yang, Jian-Hua Li, Hua Fan, Mao-Xia Yang, Yan Fu, Su-Ping Wei, Zhi-Na He, Xue-Lian Zhang, Shen-Yuan Yuan

**Affiliations:** ^1^Sanlitun Community Health Service Center, Beijing, China; ^2^Medical Records and Statistics Department, Beijing Ditan Hospital, Capital Medical University, Beijing, China; ^3^Department of Endocrinology, Beijing Tongren Hospital, Capital Medical University, Beijing, China

## Abstract

**Objective:**

To investigate the effect of intensive management and achieving the target control more than 3 times on endpoint events during 9 consecutive years' annual assessment in type 2 diabetes (T2DM) patients in the Sanlitun Community Health Service Center in Beijing, including blood glucose, blood pressure, lipids profiles, and the joint target control.

**Methods:**

In Beijing Community Diabetes Study (BCDS), 224 patients with T2DM from the Sanlitun Community Health Service Center were enrolled in 2008. All patients were randomly assigned to the intensive management group (*n* = 113) and the standard management group (*n* = 113) and the standard management group (

**Results:**

During the nine-year follow-up, the abscission number was 35 (14.29%), among which 14 (12.39%) was in the intensive management group and 21 (18.92%) was in the standard management group. The incidence of diabetic retinopathy (6 cases, 5.41%) and diabetic nephropathy (13 cases, 11.71%) in the standard management group was significantly higher than that in the intensive management group (1 case, 0.88%; 5 cases, 4.42%), respectively (*P* < 0.05). However, there were no significant differences on the other endpoint events between the two groups (*P* < 0.05). However, there were no significant differences on the other endpoint events between the two groups (*P* < 0.05). However, there were no significant differences on the other endpoint events between the two groups (*P* < 0.05). However, there were no significant differences on the other endpoint events between the two groups (*P* < 0.05). However, there were no significant differences on the other endpoint events between the two groups (

**Conclusions:**

The intensive management can effectively reduce the occurrence of microvascular complications. The incidence of all-cause death and the other endpoint events decreased in T2DM patients who achieved the joint target control more than 3 times during the nine-year management, which improved survival time and life quality. This trial is registered with ChiCTR-TRC-13003978 and ChiCTR-OOC-15006090.

## 1. Introduction

Type 2 diabetes (T2DM) is a complex chronic disease, which requires continuous medical intervention. In addition to the control of blood glucose, it is more important to take comprehensive management to reduce multiple risk factors at the same time [1]. Since the first nationwide epidemiological survey of diabetes in 1980, the prevalence of diabetes has soared from 0.67 percent to 10.4 percent in 2013, higher in cities than in rural areas. According to the fourth epidemiological survey of diabetes in 2002, the prevalence of diabetes increased more than 2 times. Due to the low awareness rate, treatment rate, and compliance rate of diabetic patients in China [2], from 2002 to present, diabetic complications have sprung up in large numbers over 16 years. Although a large number of evidence-based studies have confirmed that the joint target control of blood glucose, blood pressure, blood lipid, and other risk factors is the key to the prevention and treatment of chronic complications of diabetes [3], there are few reports on the effect of intensive management and number of times achieving the target control on endpoint events in T2DM. The Beijing Community Diabetes Study (BCDS) project, which began in 2008, went deep into the districts and counties of Beijing, trained community health care personnel, and improved the comprehensive ability of community health care units for diabetes management. From the beginning of the project, 224 patients with T2DM in Sanlitun Community Health Service Center were followed up for 9 years, monitored regularly, and standardized the diabetes management process. The purpose of this study was to analyze all-cause endpoints events that occurred during 9 consecutive years of follow-up and also to explore the benefit of quality of life of patients with T2DM in urban community after management.

## 2. Subjects and Methods

### 2.1. Study Population and Intervention Methods

**Figure 1:**
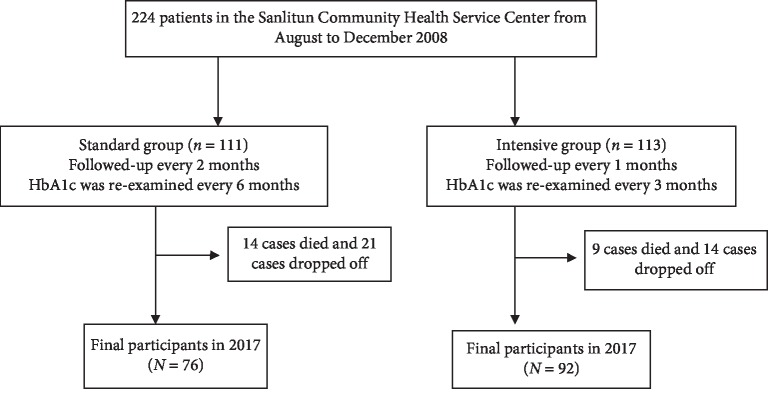
Flow chart showing the number of study participants.

In the BCDS study, two hundred twenty-four patients with T2DM were recruited from the Sanlitun Community Health Service Center from August to December 2008 according to the diagnostic criteria established by the WHO. Inclusion criteria and exclusion criteria refer to BCDS project research methods [4]. All patients were randomly divided into the intensive group (Group 1, *n* = 113) and standard group (Group 2, *n* = 111). From January 2009 to December 2017, during the 9 years of follow-up, 23 cases died and 35 cases dropped off. The total drop-off rate in 9 years was 15.63%, among which the drop-off rate of the intensive group was 12.39% and that of the standard group was 18.92% (Figure 1).

Patients in both groups were followed up and intervened according to China guideline for the prevention and treatment of T2DM [5]. The standard group was followed up every 2 months, and glycosylated hemoglobin (HbA1c) was re-examined every six months; the intensive group was followed up every month, and HbA1c was re-examined every 3 months. The patients in these two groups were evaluated once a year, including physical examination, biochemical examination, carotid ultrasound, fundus examination, electrocardiogram (ECG), HbA1c, and urinary albumin excretion rate (UAER).

The director of the endocrinology department of a top-tier hospital visited patients every week to improve the ability of general practitioners through training and tutoring. The community center was responsible for regular re-examination, collection of data, and data input and uploading.

According to China guidelines for the prevention and treatment of T2DM [6], the target control was defined as HbA1c < 7.0 mmol/L, systolic blood pressure (SBP) < 130 mmHg, diastolic blood pressure (DBP) < 80 mmHg, and low-density lipoprotein cholesterol (LDL-C) < 2.6 mmol/L (with coronary heart disease < 1.8 mmol/L). HbA1c and UAER were tested centralized by using VARIANT high pressure liquid phase instrument and the instrument of Berle Company of America in the Endocrinology Laboratory of Beijing Tongren Hospital.

The primary endpoint events include all-cause death, cardiovascular and cerebrovascular complications, stroke (cerebral infarction with limb disorder and cerebral hemorrhage), and microvascular complications. Microvascular complications include diabetic nephropathy (creatinine doubling and dialysis) and diabetic retinopathy (fundus photocoagulation and vitrectomy).

The secondary endpoint events include (1) cerebrovascular diseases, such as transient ischemic attack and subarachnoid hemorrhage, (2) cardiovascular events, such as coronary bypass graft, coronary stent, unstable angina pectoris, and heart failure hospitalization, (3) peripheral vascular disease such as peripheral vascular reconstruction, emergency thrombolytic therapy, chronic ulcer of lower extremities, and stenosis or occlusion of large vessels, (4) diabetic foot, chronic osteomyelitis, and amputation, (5) malignant tumor, (6) diabetic nephropathy such as emerging microalbuminuria or progression to >200 *μ*g/min, and (7) severe hypoglycemia.

All the abovementioned endpoint events must be supported by death certificates, medical records, diagnostic certificates, laboratory tests, and examination reports provided by the patient or his family. The photographs were uploaded to the data center and checked.

### 2.2. Statistical Analyses

SPSS 17.0 software was used to analyze the statistical data. The data with normal distribution were described by the mean ± standard deviation; the two independent samples were compared with each other by using the *t*-test. The Wilcoxon test was used to compare the difference between groups with nonnormal distribution, the frequency and rate were used to describe the counting data, the Kaplan–Meier method was used to analyze the terminal event data, and the log-rank test was used to compare the time distribution of the terminal events. If *P* < 0.05, the difference was statistically significant (note: loss interview is recorded as censored data in survival analysis, and death is recorded as censored data in analysis of nondeath endpoint events).

## 3. Results

### 3.1. Patients' Clinical Characteristics

At baseline, there were no significant differences in age, sex, body mass index (BMI), duration of diabetes, and UAER between two groups (Table 1). After 9 years of management, a total of 168 subjects were examined in 2017. Compared with the standard group, there was no significant difference in BMI, SBP, and DBP between the two groups (Table 2). The following variables reached the target control in the intensive group when compared with that of the standard group, including fasting plasma glucose (FPG), postprandial blood glucose (HPG), HbA1c, LDL-C, and UAER (Table 2).

### 3.2. Effect of Multifactorial Intervention on the Incidence of All-Cause Events

**Figure 2:**
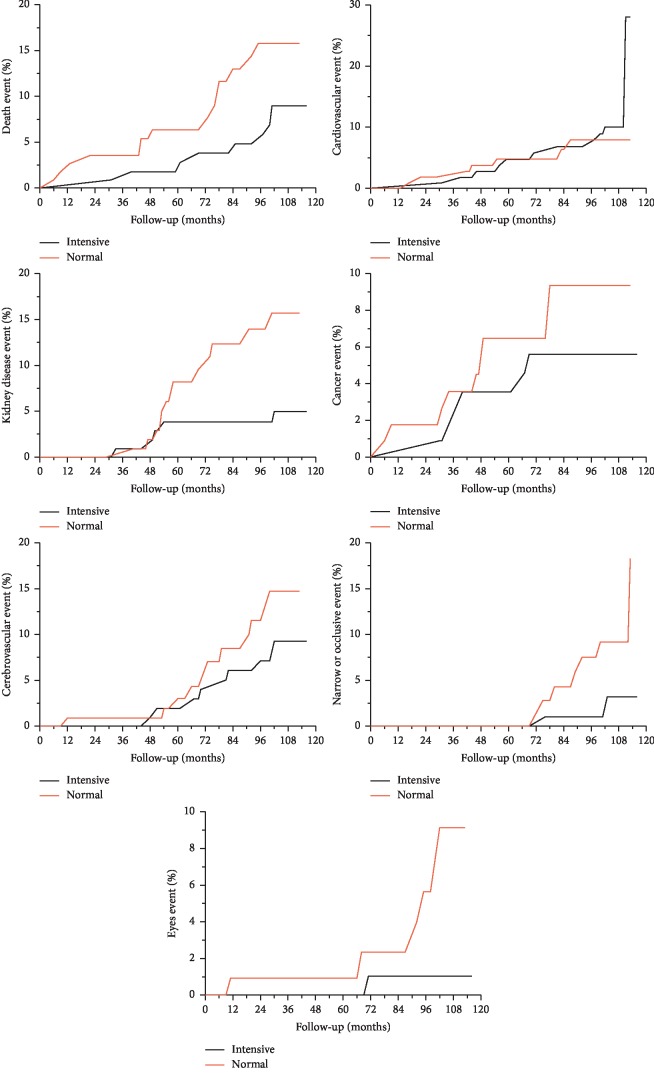
Cumulative incidences of all-cause end-point events during the follow-up between the two groups.

There were significant differences in the incidence of diabetic retinopathy and diabetic nephropathy between the two groups. There was no significant difference in the incidence of the other endpoint events between the two groups (Table 3 and Figure 2).

All-cause death rates in patients who achieved the target control (HbA1c and LDL-C) and the joint target control more than 3 times were significantly lower than those of less than 3 times. As far as death caused by cardiovascular events, cerebrovascular events, and newly onset coronary heart disease are concerned, there were no significant differences on the aforementioned endpoint events between the two groups based on target control achieved more than 3 times or not. There was less incidence of new onset cerebrovascular events, stenosis or occlusion of large arteries, and diabetic microvascular complications in patients who achieved target control (HbA1c and LDL-C) and the joint target control more than 3 times than those less than 3 times (Figure 2).

There were less incidence of microvascular events in target control in HbA1c and the joint target control more than 3 times when compared with those less than 3 times. There was significant difference in the incidence of malignant tumors between the patients reaching target control (blood pressure and joint target control) and those who not. However, no relationship between coronary heart disease and the number of joint target control was observed during the management period (Table 4).

## 4. Discussion

It is difficult for BCDS to study the effect of multifactorial intervention for 9 years in the community. Firstly, with the advancement of the urbanization process, the patients' address is constantly changing, and a large number of telephone supervision and follow-up work need the family doctor in community health service center to pay more patience and perform more meticulous work.

Secondly, diabetes is a systemic disease and needs life-long treatment. The long-term meticulous management includes examination of the main monitoring indexes and the screening of the complications, which will benefit the patients and reduce the economic burden [6, 7], as it is not conducive for self-examination and accept the management in patients. However, the medical and nursing staffs in the community health service institutions are also unstable. It needs a relatively stable management team and institutional guarantee during the management process [8]. It needs the family doctor team to cooperate with the professors from a top-tier hospital together to remind the patients to follow-up regularly. However, to get the results of the continuous management of diabetes in China, with BCDS experts and the community health service team, together for 9 years, is a magic work itself. BCDS project was in the community for 9 years, providing patients with continuous, high quality, and accessible diabetes management. There is no similar research in our country. The project has raised the level of community of T2DM management in the form of the community experts, including deputy chief physician, and 10 papers were published. The BCDS improved the quality of life in patient, greatly improved the patient's satisfaction, and constantly summarized the experience to explore a more practical approach to the management of the community T2DM.

In this study, the incidence of in all-cause death in patients with target control in blood pressure, HbA1c, LDL-C, and joint target control more than 3 times is 9 cases (6.00%), 10 cases (6.94%), 7 cases (6.73%), and 1 case (1.64%) when compared with those who were less than 3 times. There was no statistical difference in coronary heart disease between the two groups. However, Zhuangning and others in Jinsong Health Centre Community found that after 6-year intervention, there were fewer incidences of all-cause death events, cardiovascular events, and total endpoint events in patients who achieved the joint target control more than 3 times than those with the joint target control less than 3 times. Although both of these two studies were part of the BCDS study, the reasons for the different results were as follows. The average age of patients in Jinsong Health Centre Community was 64 years old; the average duration of diabetes was 8 years, while the average age of patients in the Sanlitun Health Centre Community was over 65 years old. The duration of diabetes in the intensive group was 9.0 years (13.0 ± 4.3 years) and in the standard group was 5.5 years (11.0 ± 2.0 years). It is well known that chronic complications of type 2 diabetes are directly related to age and course of disease. Of course, it is also related to the number of cases, which need to be supplemented by the conclusion of BCDS overall analysis. There were 9 cases (6.25%) of cerebrovascular disease with HbA1c target control more than 3 times, and no cerebrovascular events in patients with joint target control more than 3 times; the incidence rate was significantly lower when compared with that who did not. The symptoms of cardiovascular and cerebrovascular diseases are obvious and easy to recognize by patients.

In recent years, the government and health departments have strengthened health promotion and health education in the prevention and treatment of cardiovascular and cerebrovascular diseases. The development of interventional medicine has greatly improved the prognosis of patients with cardiovascular disease; patients are aware of and willing to accept early intervention therapy. In this study, both intensive management and standard management were based on the Chinese guidelines for the prevention and treatment of T2DM. Except for the follow-up period and monitoring frequency of the patients, the complications' monitoring and differentiation, referral, and management criteria are the same in the two groups. Therefore, there was no significant difference in the occurrence and intervention of cardiovascular endpoint events in this study. Cerebrovascular disease is more in Chinese population than coronary heart disease [9], so the main indexes are different with different standards. With the development of medical reform in China, the rehabilitation management to return to the community after special treatment of cardiovascular and cerebrovascular diseases needs more attention, which is of great significance to the secondary and tertiary prevention of cardiovascular and cerebrovascular diseases and improve patients' survival.

New-onset diabetic retinopathy and diabetic nephropathy, original diabetic retinopathy, and exacerbation of diabetic nephropathy can be classified as microvascular complications [10]. At baseline, the patients in the intensive group and the standard group had been diagnosed with diabetes for 9.0 (13.0 ± 4.3) years and 5.5 (11.0 ± 2.0) years, respectively. The HbA1c and LDL-C levels of the intensive group and standard group were 7.37 ± 1.28% vs. 7.7 ± 1.82% and 2.79 ± 0.82 vs. 2.84 ± 0.91 mmol/L, respectively. There was no statistical difference between the two groups. At this time, the oxidative stress response induced by hyperglycemia and the damage to vascular endothelium had existed for some time.

After intervention, 7 diabetic retinopathy cases occurred: 1 case (0.88%) in the intensive group and 6 cases (5.41%) in the standard group. Diabetic nephropathy occurred in 18 cases: 5 cases (4.42%) in the intensive group and 13 cases (11.71%) in the standard group. There were statistical differences between the two groups. There was no significant difference between the two groups in the occurrence of other endpoint events, including all-cause death, coronary heart disease, cerebrovascular disease, stenosis or occlusion of large arteries, and malignant tumors. The results showed that microvascular lesions were more sensitive to diabetic management.

The incidence of large artery stenosis or occlusion occurred in patients who reached the target control of blood pressure (2.67%) and HbA1c (2.08%) more than 3 times. According to joint target control more than 3 times, there were no events of stenosis or occlusion. New-onset diabetic microvascular complications significantly decreased in subjects with target control more than 3 times when compared with those less than 3 times. The more items or times of reaching the target control, the less the number of all-cause death, stenosis or occlusion of the great artery, and microvascular complications will occurr, especially the more times of reaching the standard level of HbA1c and joint target control. The importance of strengthening diabetic management for more times requires special attention of the family doctor team. In cooperation with Ophthalmology Research Institute of Beijing Tongren Hospital, the patients were examined six visual fields to check fundus problems for the first time. Because of the high visibility of ophthalmology in the whole country, the credibility of ophthalmology was relatively high.

There was no statistical difference in the control of blood pressure between groups before and after management. At the time of admission, systolic blood pressure had reached 129.03 ± 13.49 mmHg and 127.34 ± 12.53 mmHg in the intensive group and the standard group, respectively. Although the UK Prospective Diabetes Study (UKPDS) suggests that blood pressure control needs “lower as possible,” considering the average age of the patients was over 70 years old in the present study, the target control of blood pressure was defined as lower than 140/90 mmHg [11]. There was significant difference in the incidence of malignant tumors between the patients reaching target control (blood pressure and conjunctive standard) and those who not.

Malignant tumor occurrences increase in patients without reaching target control of blood pressure and the combined standard less than 3 times. The National Cancer Center analyzed the data collected by the National Cancer Registry for the registration of malignant tumors in 2013 and 2014. It is estimated that the incidence of malignant tumor in urban areas of China in 2014 is 302.13/100000 [12], and that in the elderly population in 2013 is 1029.16/100000 [13]. In this study, the incidence of malignant tumor in diabetic patients is 5.96%, which is significantly higher than that in the general population. Screening methods should be done in elderly people to diagnose and treat as early as possible.

The limitation of this study is that there is the possibility of delayed collection or omission of endpoint events in 9-year management, which may affect the accuracy of the analysis results. We try to find evidence from all possible sources in our study to determine the exact timing of the endpoint event to avoid greater bias. Because of the characteristics of the area and population in the Sanlitun community area and the small number of cases, the conclusion of this study is only of some reference significant to the urban communities with elderly people.

## Figures and Tables

**Table 1:** Comparison of clinical characteristics between the intensive group and the standard group at baseline (2008).

Characteristics	Intensive group	Standard group	Statistical quantity	*P* value
	(*n* = 113)	(*n* = 111)	(*t*/*χ*^2^)	
Age (years)	66.46 ± 8.47	65.55 ± 9.88	0.74 (*t*)	0.46
Gender				
Male, *n*	39	39	−0.00 (*χ*^2^)	1.00
Female, *n*	74	72		
Duration of diabetes (years)	9.0 (4.3, 13.0)	5.5 (2.0, 11.0)	1.49 (*t*)	0.14
Smoker				
Yes	11	7	0.49 (*χ*^2^)	0.49
No	102	104		
Body mass index (kg/m^2^)	25.12 ± 3.31	25.32 ± 3.57	−0.42 (*t*)	0.68
Systolic blood pressure (mmHg)	129.03 ± 13.49	127.34 ± 12.53	0.96 (*t*)	0.34
Diastolic blood pressure (mmHg)	76.59 ± 7.40	77.9 ± 6.43	−1.40 (*t*)	0.16
FPG (mmol/L)	7.69 ± 2.46	8.18 ± 2.77	−1.36 (*t*)	0.17
HPG (mmol/L)	9.19 ± 2.90	9.82 ± 3.22	−1.30 (*t*)	0.20
HbA1c (%)	7.37 ± 1.28	7.78 ± 1.82	−1.92 (*t*)	0.06
LDL-cholesterol (mmol/l)	2.79 ± 0.82	2.84 ± 0.91	−0.45 (*t*)	0.65
UAER (*μ*g/min)	6.25 (4.69, 12.96)	6.78 (5.21, 14.17)	−1.05 (*t*)	0.29

*N* = number of individuals. Values are expressed as mean ± SD, median (Q1, Q3), or number (%). *P* values indicate statistical significance of the differences among the three groups. BMI, body mass index; SBP, systolic blood pressure; DBP, diastolic blood pressure; LDL-C, low-density lipoprotein cholesterol; FPG, fasting plasma glucose; HPG, 2-hour postprandial blood glucose; HbA1c, hemoglobin A1c; UAER, urinary albumin excretion rate.

**Table 2:** Comparison of clinical characteristics between the intensive group and the standard group at the end of follow-up (2017).

Characteristic	Intensive group	Standard group	Statistical quantity	*P* value
	(*n* = 92)	(*n* = 96)	(*t*/*χ*^2^)	
Body mass index (kg/m^2^)	24.93 ± 2.74	25.79 ± 3.70	−1.68 (*t*)	0.09
Systolic blood pressure (mmHg)	126.95 ± 9.93	127.75 ± 11.30	−0.49 (*t*)	0.62
Diastolic blood pressure (mmHg)	71.47 ± 7.90	71.04 ± 7.43	0.36 (*t*)	0.72
FPG (mmol/L)	7.25 ± 1.73	8.07 ± 2.13	−2.78 (*t*)	0.01
HPG (mmol/L)	8.34 ± 1.52	10.03 ± 3.13	−4.3 (*t*)	0.01
HbA1c (%)	6.87 ± 1.14	7.43 ± 1.39	−2.81 (*t*)	0.01
LDL-cholesterol (mmol/l)	2.50 ± 1.03	2.89 ± 0.86	−2.64 (*t*)	0.01
UAER (*μ*g/min)	6.32 (0.37, 275.33)	8.69 (2.08, 524.28)	−2.55 (*t*)	0.01

*N* = number of individuals. Values are expressed as mean ± SD, median (Q1, Q3), or number (%). *P* values indicate statistical significance of the differences among the three groups. BMI, body mass index; SBP, systolic blood pressure; DBP, diastolic blood pressure; LDL-C, low-density lipoprotein cholesterol; FPG, fasting plasma glucose; HPG, 2-hour postprandial blood glucose; HbA1c, hemoglobin A1c; UAER, urinary albumin excretion rate.

**Table 3:** The incidence of endpoint events between the two groups.

End-point events	Intensive group	Standard group	Log-rank	*P* value
	(*n* = 113)	(*n* = 111)		
All-cause death	9 (7.96)	14 (12.61)	2.51	0.11
Coronary heart disease	11 (9.73)	7 (6.31)	0.43	0.51
Cerebrovascular disease	9 (7.64)	11 (9.91)	1.26	0.26
Stenosis or occlusion of large arteries	3 (2.65)	7 (6.31)	3.15	0.08
Diabetic retinopathy	1 (0.88)	6 (5.41)	5.89	0.02
Diabetic nephropathy	5 (4.42)	13 (11.71)	5.44	0.02
Malignant tumor	6 (5.31)	9 (8.11)	1.05	0.31

**Table 4:** Comparison of the endpoint events in patients who achieved the target control (HbA1c, blood pressure, LDL-C, and the joint target control) >3 times and <3 times.

Clinical characteristics	*n*	All-cause death	Coronary heart disease	Cerebrovascular disease	Stenosis or occlusion of large arteries	Diabetic microvascular complications	Malignant tumor
*Target control in blood pressure*							
≥3 times	150	9 (6.00)	13 (8.67)	14 (9.33)	4 (2.67)	18 (12.00)	6 (4.00)
<3 times	74	14 (18.92)	5 (6.76)	6 (8.11)	6 (8.11)	7 (9.46)	9 (12.16)
*χ* ^2^		13.49	0.01	0.21	7.93	0.11	6.86
*P*		0.00	0.92	0.65	0.01	0.75	0.01

*Target control in HbA1c*							
≥3 times	144	10 (6.94)	10 (6.94)	9 (6.25)	3 (2.08)	13 (9.03)	7 (4.86)
<3 times	80	13 (16.25)	8 (10.00)	11 (13.75)	7 (8.75)	12 (15.00)	8 (10.00)
*χ* ^2^		7.77	2.04	7.17	9.65	4.39	3.21
*P*		0.01	0.15	0.01	0.00	0.04	0.07

*Target control in LDL-C*							
≥3 times	104	7 (6.73)	10 (9.62)	8 (7.69)	4 (3.85)	10 (9.62)	4 (3.85)
<3 times	120	16 (13.33)	8 (6.67)	12 (10.00)	6 (5.00)	15 (12.50)	11 (9.17)
*χ* ^2^		4.9	0.06	2.24	1.34	2.15	3.7
*P*		0.03	0.81	0.13	0.25	0.14	0.06

*Joint target control*							
≥3 times	61	1 (1.64)	6 (9.84)	0 (0.00)	0 (0.00)	3 (4.92)	1 (1.64)
<3 times	163	22 (13.50)	12 (7.36)	20 (12.27)	10 (6.13)	22 (13.50)	14 (8.59)
*χ* ^2^		8.63	0	11.35	6.15	5.66	4.12
*P*		0.00	0.96	0.00	0.01	0.02	0.04

## Data Availability

The data used to support the findings of this study are included within the article.
